# Ravulizumab in Atypical Hemolytic Uremic Syndrome: An Analysis of 2-Year Efficacy and Safety Outcomes in 2 Phase 3 Trials

**DOI:** 10.1016/j.xkme.2024.100855

**Published:** 2024-06-14

**Authors:** Bradley P. Dixon, David Kavanagh, Alvaro Domingo Madrid Aris, Brigitte Adams, Hee Gyung Kang, Edward Wang, Katherine Garlo, Masayo Ogawa, Praveen Amancha, Sourish Chakravarty, Nils Heyne, Seong Heon Kim, Spero Cataland, Sung-Soo Yoon, Yoshitaka Miyakawa, Yosu Luque, Melissa Muff-Luett, Kazuki Tanaka, Larry A. Greenbaum

**Affiliations:** 1 Renal Section, Department of Pediatrics, University of Colorado School of Medicine, Aurora, CO; 2 National Renal Complement Therapeutics Centre, Newcastle upon Tyne Hospitals NHS Foundation Trust and Translational and Clinical Research Institute, Newcastle University, Newcastle upon Tyne, United Kingdom; 3 Children’s Nephrology and Renal Transplantation Service, Children’s Maternity Hospital Sant Joan de Déu, University of Barcelona, Barcelona, Spain; 4 Department of Pediatric Nephrology, Children’s Hospital Queen Fabiola, Université libre de Bruxelles, Brussels, Belgium; 5 Division of Pediatric Nephrology, Department of Pediatrics, Seoul National University College of Medicine, Seoul, Republic of Korea; 6 Alexion, AstraZeneca Rare Disease, Boston, MA; 7 Section of Nephrology and Hypertension, Department of Internal Medicine IV, University Hospital Tübingen, Tübingen, Germany; 8 Department of Pediatrics, Pusan National University Children’s Hospital, Yangsan, Republic of Korea; 9 Division of Hematology, The Ohio State University Medical Center, Columbus, OH; 10 Department of Internal Medicine, Seoul National University Hospital, Seoul, Republic of Korea; 11 Department of Hematology, Saitama Medical University, Saitama, Japan; 12 Renal Intensive Care Unit, Nephrology Department, Tenon Hospital, Assistance Publique-Hôpitaux de Paris, Sorbonne Université, Paris, France; 13 Division of Pediatric Nephrology, University of Nebraska Medical Center, Omaha, NE; 14 Department of Nephrology, Aichi Children’s Health and Medical Center, Aichi, Japan; 15 Division of Pediatric Nephrology, Emory University School of Medicine and Children’s Healthcare of Atlanta, Atlanta, GA

**Keywords:** Adult, atypical hemolytic uremic syndrome, complement C5 inhibitor, efficacy, hematology, nephrology, pediatric, ravulizumab, safety, thrombotic microangiopathy

## Abstract

**Rationale & Objective:**

Atypical hemolytic uremic syndrome (aHUS) is a rare form of thrombotic microangiopathy (TMA) caused by complement dysregulation. Ravulizumab is a C5i approved for the treatment of aHUS. This analysis assessed long-term outcomes of ravulizumab in adults and pediatric patients with aHUS.

**Study Design:**

This analysis reports 2-year data from 2 phase 3, single-arm studies.

**Setting & Participants:**

One study included C5i-naïve adults (NCT02949128), and the other included 2 cohorts of pediatric patients (C5i-naïve and those who switched to ravulizumab from eculizumab [pediatric switch patients]; NCT03131219).

**Exposure:**

Patients received intravenous ravulizumab every 4-8 weeks, with the dose depending on body weight.

**Outcomes:**

The primary endpoint in the studies of C5i-naïve patients was complete TMA response, which consisted of platelet count normalization, lactate dehydrogenase normalization, and ≥25% improvement in serum creatinine concentrations from baseline, at 2 consecutive assessments ≥4 weeks apart.

**Analytical Approach:**

All analyses used descriptive statistics. No formal statistical comparisons were performed.

**Results:**

In total, 86 and 92 patients were included in efficacy and safety analyses, respectively. Complete TMA response rates over 2 years were 61% and 90% in C5i-naïve adults and pediatric patients, respectively. The median increase in estimated glomerular filtration rate from baseline was maintained over 2 years in C5i-naïve adults (35 mL/min/1.73 m^2^) and pediatric patients (82.5 mL/min/1.73 m^2^). Most adverse events and serious adverse events occurred during the first 26 weeks. No meningococcal infections were reported. Improvement in the Functional Assessment of Chronic Illness Therapy – Fatigue score achieved by 26 weeks was maintained over 2 years.

**Limitations:**

Limitations were the small sample of pediatric switch patients and limited availability of genetic data.

**Conclusions:**

Long-term treatment with ravulizumab is well tolerated and associated with improved hematologic and renal parameters and quality of life in adults and pediatric patients with aHUS.

## Plain-Language Summary

This research tested a drug called ravulizumab for the treatment of atypical hemolytic uremic syndrome (aHUS). aHUS is a rare disease that causes clots in tiny blood vessels. This can damage the kidneys and other organs. We analyzed data from 2 clinical trials in which children and adults with aHUS received ravulizumab through a tube placed in a vein (intravenous line). They received ravulizumab every 4-8 weeks depending on their weight. We found that treating patients for 2 years with ravulizumab was associated with improved blood health, kidney function, and quality of life and was well tolerated. These results support ravulizumab as a long-term treatment for people with aHUS.

Atypical hemolytic uremic syndrome (aHUS) is a rare thrombotic microangiopathy (TMA) characterized by microangiopathic hemolytic anemia, thrombocytopenia, and acute kidney injury.^1^, ^2^, ^3^ aHUS may progress to chronic kidney disease and permanent kidney failure, as well as damage other organ systems, leading to severe morbidity or death.^4^^,^^5^

The complement C5 inhibitor (C5i) eculizumab has been shown to be effective for the treatment of patients with aHUS since its approval in 2011.^6^^,^^7^ However, it requires infusions every 2 weeks for patients who weigh ≥10 kg, which may impose a treatment burden on patients, caregivers, and the health care system.^1^^,^^8^, ^9^, ^10^, ^11^ Ravulizumab, a next-generation terminal C5i, was designed by targeted modification of eculizumab to enhance antibody recycling and attenuate target-mediated drug disposition to achieve an extended half-life and improve weight-based dosing.^12^ Ravulizumab has been approved for the treatment of aHUS among other indications in the United States, Europe, and Japan and requires maintenance doses every 4 or 8 weeks, depending on body weight.^13^, ^14^, ^15^ Ravulizumab provides immediate, complete, and sustained C5 inhibition and has demonstrated favorable efficacy and tolerability in 2 phase 3 studies in adult and pediatric patients with aHUS.^16^, ^17^, ^18^, ^19^

The objective of this analysis was to assess the long-term efficacy and safety of ravulizumab over 2 years for the management of aHUS.

## Methods

**Figure 1:**
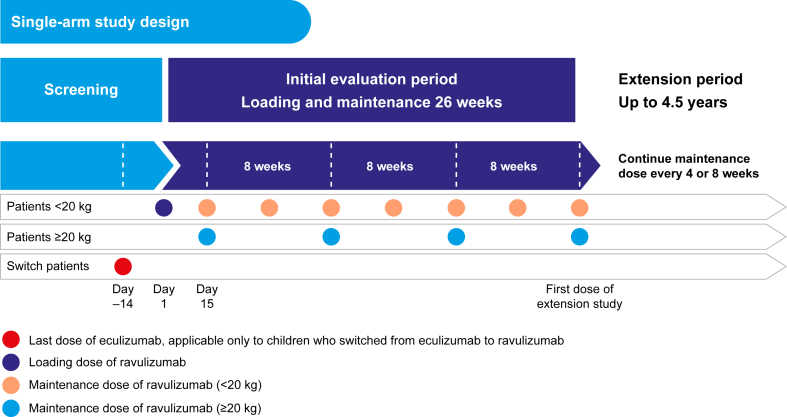
Study design.

This analysis reports 2-year data from the phase 3, single-arm, multicenter studies that were conducted to assess the efficacy and safety of intravenous ravulizumab administered every 4-8 weeks, depending on body weight, in patients with aHUS. One study was in adults (NCT02949128)^17^^,^^18^ and the other study was in the following 2 cohorts of pediatric patients: (1) C5i-naïve; (2) those who switched to ravulizumab from eculizumab (NCT03131219; pediatric switch patients)^16^^,^^19^ (Fig 1). After the 26-week primary evaluation period in each study, participants could enter an extension period and receive ravulizumab until product registration or approval (in accordance with country-specific regulations) or for up to 4.5 years, whichever occurred first.

The full inclusion and exclusion criteria for both studies have been reported previously.^16^, ^17^, ^18^, ^19^ Briefly, inclusion criteria were adult or pediatric patients with aHUS naïve to C5i who demonstrated clinical features of TMA, including thrombocytopenia, as defined by a platelet count of <150,000/μL, hemolysis as defined by lactate dehydrogenase (LDH) >1.5× the upper limit of normal, and kidney injury as defined by serum creatinine criteria less than or equal to the upper limit of normal for adults and ≥97.5 percentile for age at screening for pediatric patients. Alternative causes of TMA, including hemolytic uremic syndrome caused by Shiga toxin-producing *Escherichia coli* or thrombotic thrombocytopenic purpura*,* were excluded by laboratory testing.^16^, ^17^, ^18^, ^19^ Pediatric switch patients were required to have a previous diagnosis of aHUS and clinical evidence of response to eculizumab indicated by stable TMA parameters (including LDH <1.5× the upper limit of normal, platelet count of ≥150,000/μL, and estimated glomerular filtration rate [eGFR] of >30 mL/min/1.73 m^2^ using the modified Schwartz equation).^20^

The safety analysis set included all patients who received ≥1 infusion of ravulizumab in the initial evaluation period. The full analysis set was used for analyses of efficacy, demographics, and baseline characteristics and included all patients with confirmed study eligibility and who received ≥1 infusion of ravulizumab in the primary evaluation period, including those later discontinued, and ≥1 post-baseline efficacy assessment.

The primary endpoint in the studies of patients who were C5i-naïve was complete TMA response, which consisted of platelet count normalization, LDH normalization, and ≥25% improvement in serum creatinine concentrations from baseline, at 2 consecutive assessments ≥4 weeks apart.^16^, ^17^, ^18^ Secondary efficacy endpoints among all cohorts included dialysis requirement status, change from baseline in eGFR and chronic kidney disease stage, hematologic parameters (platelets, LDH, and hemoglobin), quality of life (measured by the pediatric Functional Assessment of Chronic Illness Therapy – Fatigue [FACIT-Fatigue] scale), and TMA parameters in patients who discontinued treatment during the extension period but remained in the study. Pharmacodynamic endpoints included changes in serum-free C5 concentrations (a measure of terminal complement inhibition) over time. Pharmacodynamic analyses were performed in all patients with evaluable data who had received ≥1 dose of ravulizumab.

Descriptive statistics were used for all analyses. No formal statistical comparisons were performed. For TMA response data, 95% confidence intervals (CIs) for the proportion are based on exact confidence limits using the Clopper-Pearson method.

## Results

### Patient Disposition

**Figure 2:**
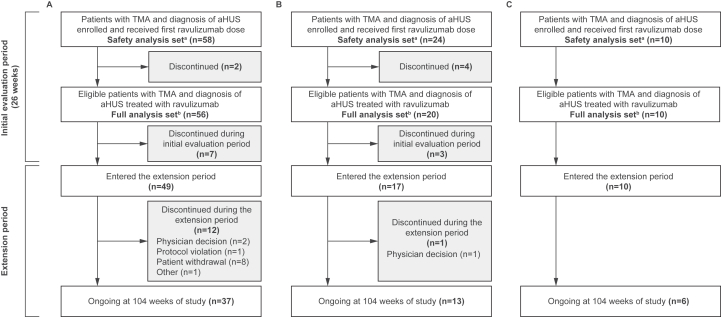
Patient disposition in adults naïve to C5i treatment (A), pediatric patients naïve to C5i treatment (B), and pediatric switch patients (C). ^a^The safety analysis set was used for safety analyses and included all patients who received ≥1 infusion of ravulizumab in the initial evaluation period. ^b^The full analysis set was used for analyses of efficacy, demographics, and baseline characteristics and included all patients with confirmed study eligibility and who received ≥1 infusion of ravulizumab in the initial evaluation period, including those later excluded, and ≥1 post-baseline efficacy assessment. Abbreviations: aHUS, atypical hemolytic uremic syndrome; C5i, complement C5 inhibitor; TMA, thrombotic microangiopathy.

The safety analysis set included 92 patients (C5i-naïve adults: n=58; C5i-naïve pediatric patients: n=24; pediatric switch patients: n=10), and the full analysis set included 86 patients (C5i-naïve adults: n=56; C5i-naïve pediatric patients: n=20; pediatric switch patients: n=10) (Fig 2). At 2-year follow-up, 13 patients had discontinued the extension phase completely. The reasons for patient discontinuation among C5i-naïve adults included patient withdrawal (n=8), physician decision (n=2), protocol violation (n=1), and other (n=1). Among C5i-naïve pediatric patients, 1 patient discontinued from the extension period because of physician decision; no patients discontinued among pediatric switch patients.

### Baseline Characteristics

Baseline characteristics of patients in the overall study populations are summarized in Table 1. As reported previously,^16^^,^^17^^,^^19^ among C5i-naïve adults with available genetic data (mutations in ≥1 complement genes or complement factor H autoantibodies; n=45), a complement abnormality (pathogenic variant, likely pathogenic variant, or complement factor H autoantibodies) was detected in 14 patients (31%), of whom 12 (27%) entered the extension period (Table S1).^17^ Among C5i-naïve pediatric patients with available data (n=17), 11 patients (65%) had a complement abnormality, of whom 10 (59%) entered the extension (Table S1).^16^ Similarly, among pediatric switch patients with available data (n=10), 6 (60%) had a complement abnormality, and all continued into the extension (Table S1).^19^

**Table 1:** Demographics and Baseline characteristics (Full Analysis Set)

	C5i-Naïve Adults (n=56)	C5i-Naïve Pediatric Patients (n=20)	Pediatric Switch Patients (n=10)
Sex, female, n (%)	37 (66%)	12 (60%)	1 (10%)
Race, n (%)^a^			
White	29 (52%)	11 (55%)	5 (50%)
Asian	15 (27%)	5 (25%)	4 (40%)
Black or African American	2 (4%)	3 (15%)	1 (10%)
American Indian or Alaskan native	1 (2%)	1 (5%)	0 (0%)
Other	1 (2%)	0 (0%)	0 (0%)
Unknown	8 (14%)	1 (5%)	0 (0%)
Age at first aHUS symptom, median (range), y	40 (9-77)	4 (1-15)	5 (<1 to 8)
Age at first infusion, median (range), y	40 (20-77)	5 (1-17)	13 (1-16)
Body weight at time of first ravulizumab infusion, median (range), kg	68 (46-112)	16 (8-69)	48 (9-69)
Received a kidney transplant before study entry, n (%)	8 (14%)	1 (5%)	1 (10%)
Received dialysis within 5 days before first ravulizumab infusion, n (%)	29 (52%)	7 (35%)	0 (0%)
Received PE/PI before first ravulizumab infusion and related to current TMA, n (%)^b^	48/58 (83%)	6/24 (25%)	0 (0%)
In intensive care unit at screening, n (%)	27/53 (51%)^c^	7 (35%)	NA

Abbreviations: aHUS, atypical hemolytic uremic syndrome; C5i, complement C5 inhibitor; NA, not applicable; PE/PI, plasma exchange/plasma infusion; TMA, thrombotic microangiopathy.

a Patients may select multiple races.

b Safety analysis set.

c Based on the total number of patients who had any emergency room visits or hospitalizations because of aHUS before start of screening. (n=53).

### Hematologic and Kidney Function Outcomes

**Figure 3:**
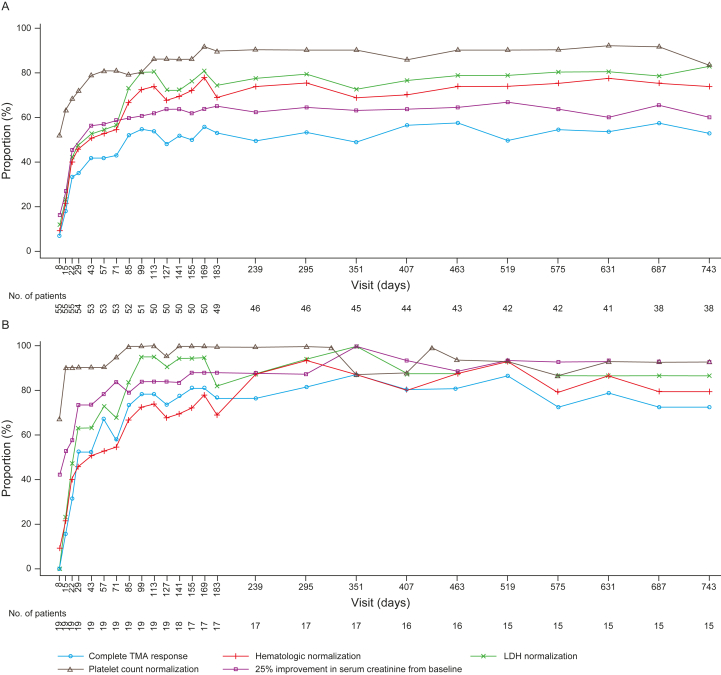
Complete TMA response and its components status over time in adults (A) and pediatric patients (B) naïve to C5i treatment up to 2 years. The criteria for complete TMA response were normalization of platelet count, normalization of LDH, and ≥25% improvement in serum creatinine concentrations from baseline. A patient was in the analysis for a specific post-baseline time point if it was possible for the result at that time point to be confirmed. Hematologic normalization included normalization of platelet count and normalization of LDH. Platelet values obtained from the day of a blood transfusion of platelets through 3 days after the transfusion were excluded from all analyses. All serum creatinine values obtained while a patient was on dialysis were excluded from all analyses. When a patient was on dialysis at baseline, then the first valid creatinine value to be used as the baseline value was the first assessment ≥6 days post-dialysis. If a patient was on dialysis during the entire 26-week initial evaluation period, then the baseline creatinine was not calculated. Data from days with ≤1 patient are not shown. Abbreviations: LDH, lactate dehydrogenase; TMA, thrombotic microangiopathy.

**Figure 4:**
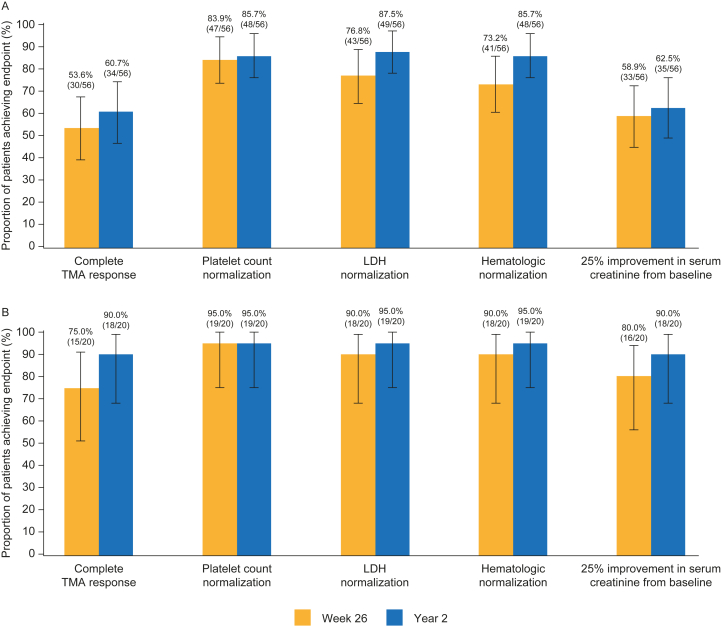
Complete TMA response and its components status over time in adults (A) and pediatric patients (B) naïve to C5i treatment at 26 weeks and 2 years. 95% confidence intervals for the proportion were based on exact confidence limits using the Clopper-Pearson method. The criteria for complete TMA response were normalization of platelet count, normalization of LDH, and ≥25% improvement in serum creatinine concentrations from baseline. Patients met all complete TMA response criteria concurrently, and each criterion was met at 2 separate assessments obtained at least 4 weeks (28 days) apart and any measurement in between. The proportion of complete TMA response was based on the responders among treated patients. Hematologic normalization included normalization of platelet count and normalization of LDH. Platelet values obtained from the day of a blood transfusion of platelets through 3 days after the transfusion were excluded from all analyses. All serum creatinine values obtained while a patient was on dialysis were excluded from all analyses. When a patient was on dialysis at baseline, then the first valid creatinine value used as the baseline value was the first assessment ≥6 days post-dialysis. If a patient was on dialysis during the entire 26-week initial evaluation period, then the baseline creatinine was not taken into consideration. Abbreviations: LDH, lactate dehydrogenase; TMA, thrombotic microangiopathy.

Complete TMA response was achieved in 34 C5i-naïve adults (61%; 95% CI, 47%-74%) and 18 C5i-naïve pediatric patients (90%; 95% CI, 68%-99%). Overall, among C5i-naïve adults, 86% achieved platelet count normalization, 88% achieved LDH normalization, 63% achieved ≥25% improvement in serum creatinine concentrations, and 86% achieved hematologic normalization over 2 years of treatment (Figs 3 and 4; Table S2). Among C5i-naïve pediatric patients, 95% achieved platelet count normalization, LDH normalization, and hematologic normalization, with 90% achieving ≥25% improvement in serum creatinine concentrations (Figs 3 and 4; Table S2). Most C5i-naïve adults achieved complete TMA response at week 26, which slightly increased over 2 years (54%; 95% CI, 40%-68% vs 61%; 95% CI, 47%-74%).^18^ This was also observed among C5i-naïve pediatric patients (75%; 95% CI, 51%-91% vs 90%; 95% CI, 68%-99%).^16^

The proportion of patients achieving platelet count normalization between week 26 and 2 years was maintained among C5i-naïve adults (84%; 95% CI, 73%-94% vs 86%; 95% CI, 76%-96%) and C5i-naïve pediatric patients (95%; 95% CI, 75%-100% for both time points). Among patients entering the extension period with available data (n=48), 4 C5i-naïve adults had ongoing thrombocytopenia (platelet count range of 86,000-143,000/μL). No pediatric patients entered the extension period with ongoing thrombocytopenia.

Because the aim of this study was to investigate the efficacy and safety of ravulizumab, clinical TMA relapse was not a defined endpoint, so it was not possible to determine the rate of TMA relapse in patients who discontinued during follow-up. However, during the 8-week safety follow-up telephone call after the last patient visit, no patients reported a relapse, or an adverse event (AE) considered to be related to relapse. One adult patient, who previously met the complete TMA response criteria while receiving therapy, discontinued treatment by choice at the completion date of the initial evaluation period.^17^ From genetic analysis, the patient did not have a pathogenic variant.

**Figure 5:**
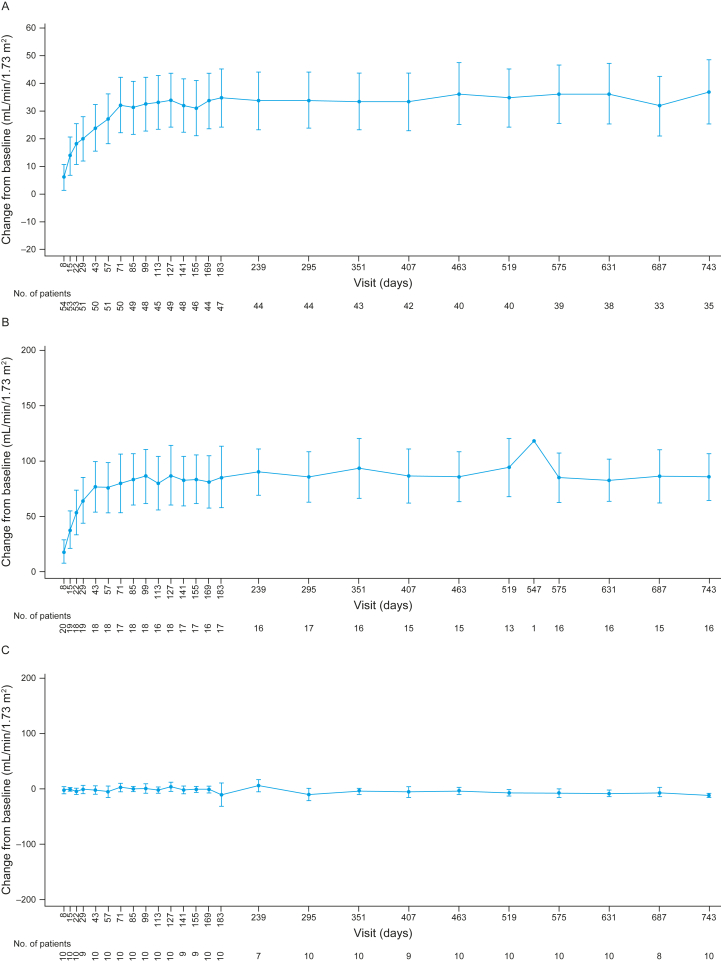
Mean observed changes in eGFR up to 2 years for adults naïve to C5i treatment (A), pediatric patients naïve to C5i treatment (B), and pediatric switch patients (C). Values indicate the mean and 95% confidence intervals. Baseline value is defined as the average of the values from the assessments performed before the first study drug infusion (these can include results from screening and the day 1 visit). 10 mL/min/1.73 m^2^ for eGFR is imputed for patients requiring dialysis for acute kidney injury. Abbreviation: eGFR, estimated glomerular filtration rate.

Kidney function improvements were maintained during this 2-year follow-up, with the median change in eGFR from baseline sustained in adults (35 mL/min/1.73 m^2^) and C5i-naïve pediatric patients (82.5 mL/min/1.73 m^2^; Fig 5A and B; Table S2). Of the 29 C5i-naïve adults on dialysis at baseline, 17 discontinued dialysis (58.6%) during the initial evaluation period.^17^ Of the 18 patients available at the 2-year visit (day 743), 12 had discontinued dialysis (66.7%). All C5i-naïve pediatric patients who received dialysis at baseline had discontinued dialysis by year 2 (Table S2). In pediatric switch patients, a median (range) change from baseline in eGFR of –13 mL/min/1.73 m^2^ (–20 to –2) was observed over 2 years (Fig 5C). Generally, the improvement in eGFR achieved with eculizumab was maintained after switching to ravulizumab, and the decrease in average appears to be driven by a few of the 10 patients (Fig S1). The largest decreases in eGFR were associated with patient 2 (day 180, eGFR 40 mL/min/1.73 m^2^) and patient 7 (day 295, eGFR 87 mL/min/1.73 m^2^); however, their LDH (177 and 188 U/L, respectively) and platelet count (196,000 and 328,000/μL, respectively) on those days remained in the normal range, likely ruling out TMA recurrence. The eGFR levels of both patients subsequently improved (patient 2, day 295, eGFR 115 mL/min/1.73 m^2^; patient 7, day 351, eGFR 123 mL/min/1.73 m^2^). The statistical significance of the overall change was not evaluated.

### Quality of Life

The improvement in FACIT-Fatigue score achieved in the initial 26-week evaluation period was maintained over 2 years, with mean observed scores plateauing between 40 and 50 (Fig S2). C5i-naïve adults and pediatric patients achieved similar FACIT-Fatigue scores to pediatric patients who switched to ravulizumab.

### Safety

All C5i-naïve adults experienced an AE over 2 years of treatment with ravulizumab, with most AEs being grade ≤3 (Table 2). Of 58 patients, 36 (62%) experienced a serious AE (SAE) (Table 2). There were 71 SAEs during the first 26 weeks of the study and 49 SAEs during the subsequent period up to 2 years.^18^ Overall, 21 patients (36%) experienced treatment-related AEs, and 3 patients (5%) discontinued the study because of AEs. All discontinuations occurred during the initial evaluation period.^18^

**Table 2:** Summary of Adverse Events Reported in Adults and Pediatric Patients Naïve to C5i Treatment up to 2 Years (Safety Analysis Set)

	Adults Naïve to C5i Treatment (n=58)		Pediatric Patients Naïve to C5i Treatment (n=24)	
	n (%)	Events	n (%)	Events
Patients with any AE	58 (100%)	1153	23 (96%)	473
AE severity				
Grade 1	56 (97%)	621	22 (92%)	299
Grade 2	49 (85%)	327	18 (75%)	135
Grade 3	34 (59%)	172	12 (50%)	38
Grade 4	15 (26%)	29	1 (4%)	1
Grade 5	3 (5%)	3	0 (0%)	0
Patients with any SAE	36 (62%)	120	16 (67%)	42
Patients with any treatment-related AE	21 (36%)	74	12 (50%)	47
Study discontinuation because of AE	3 (5%)	3	2 (8%)	3
Death because of AE^a^	3 (5%)	3	0 (0%)	0
Meningococcal infections	0 (0%)	0	0 (0%)	0

Abbreviations: AE, adverse event; C5i, complement C5 inhibitor; SAE, serious adverse event.

a Septic shock (n=2 events); cerebral hemorrhage (n=1 event).^18^

In C5i-naïve pediatric patients, 23 (96%) experienced an AE over 2 years of treatment with ravulizumab, and 16 (67%) experienced an SAE (Table 2). There were 31 SAEs during the first 26 weeks of the study and 11 SAEs during the subsequent period up to 2 years.^16^ All pediatric switch patients experienced an AE over 2 years of treatment, and 1 (10%) experienced an SAE (Table 3). No SAEs were reported among pediatric switch patients between week 26 and 2 years (n=1, 5 events).^19^ Most or all AEs were grade ≤3 for C5i-naïve pediatric patients and pediatric switch patients (Table 2). In the switch cohort, there were no discontinuations because of AEs.

**Table 3:** Summary of Adverse Events Reported in Pediatric Switch Patients up to 2 Years (Safety Analysis Set)

	Pediatric Switch Patients (N=10)	
	n (%)	Events
Patients with any AE	10 (100%)	91
AE severity		
Grade 1	8 (80%)	30
Grade 2	8 (80%)	55
Grade 3	1 (10%)	6
Grade 4	0 (0%)	0
Grade 5	0 (0%)	0
Patients with any SAE	1 (10%)	5
Patients with any treatment-related AE	2 (20%)	4
Study discontinuation because of AE	0 (0%)	0
Death because of AE	0 (0%)	0
Meningococcal infections	0 (0%)	0

Abbreviations: AE, adverse event; SAE, serious adverse event.

A summary of AEs is presented in Tables S3 and S4. The most common AEs were headache (n=23, 40%) and diarrhea (n=20, 35%) for C5i-naïve adults; pyrexia (n=13, 54%), diarrhea (n*=*8, 33%), and vomiting (n=8, 33%) for C5i-naïve pediatric patients; and upper respiratory tract infection (n=4, 40%), oropharyngeal pain (n=3; 30%), and pharyngitis (n=3, 30%) for pediatric switch patients.

No patients had a meningococcal, pneumococcus, or hemophilus influenzae type b infection during the 2-year follow-up period, and no patients died between 26 weeks and 2 years of treatment. During the 2-year analysis, 3 patients (5%) experienced an AE of sepsis (all were C5i-naïve adults), and 26 patients (28%) experienced an SAE related to infections and infestations (C5i-naïve adults: n=16; C5i-naïve pediatric patients: n=9; pediatric switch patients: n=1) (Table S5).

### Pharmacodynamics

**Figure 6:**
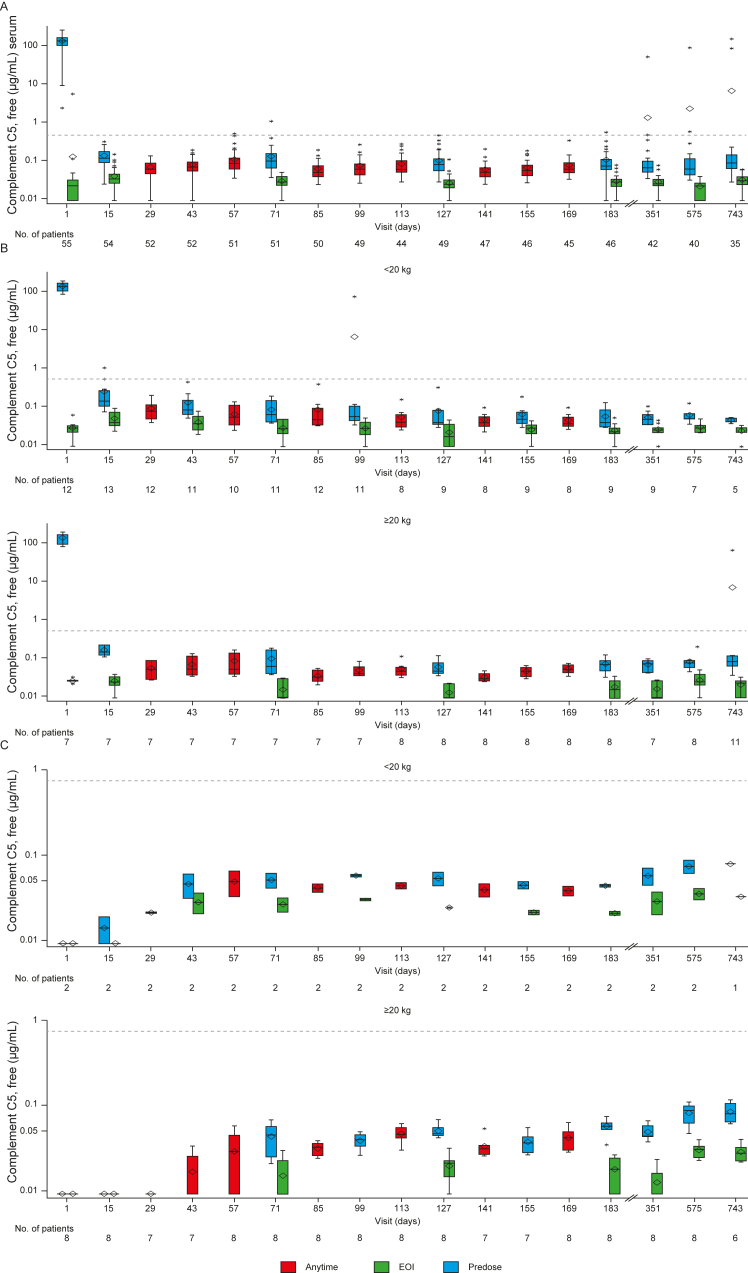
Free C5 concentrations up to 2 years for adults naïve to C5i treatment (A), pediatric patients naïve to C5i treatment (B), and pediatric switch patients (C). Dashed horizontal lines indicate serum-free C5 concentration of 0.5 μg/mL to denote the threshold for complete terminal complement inhibition. The horizontal line in the middle of each box indicates the median, a diamond indicates the mean, and the top and the bottom borders of the box mark the 75th and 25th percentiles, respectively. The whiskers represent the highest and lowest values within 1.5 times the interquartile range from the lower quartile and upper quartile. Outliers are represented by an asterisk beyond the whiskers. In B and C, patients are grouped based on the dose regimen decision day (patients on 4- or 8-week schedules). During the initial evaluation period, dose regimen decision days were screening, day 8, day 57, and day 113. During the extension period, the dose of ravulizumab was based on the patient’s body weight on the preceding decision day. For free C5 values below the limit of quantitation, LLOQ/2 = 0.00915 μg/mL was used. Abbreviation: EOI, end of infusions; LLOQ, lower limit of quantitation.

Ravulizumab treatment resulted in complete, rapid terminal complement inhibition as measured by serum-free C5 concentrations <0.5 μg/mL in the initial evaluation period, which was sustained over 2 years (Fig 6). Median (interquartile range) free C5 levels remained consistently below the threshold of 0.5 μg/mL in all cohorts.

Exceptions, in which serum-free C5 concentrations post-loading doses exceeded 0.5 μg/mL, occurred for 8 C5i-naïve adults with 12 excursion events (n=6, 1 event; n=2, 3 events; Fig 6A). Among these 12 events, 2 patients had 1 event each that did not coincide with low serum ravulizumab concentrations. Both events were observed during the primary evaluation period; one at day 1 was deemed an outlier pharmacodynamic sample, the other was associated with a patient with protocol deviations (who received plasma exchange/plasma infusion 3 times within 2 weeks before the excursion event). The remaining 10 events were associated with low serum ravulizumab concentrations (<175 μg/mL); 2 related to an extended period (>24 weeks) of treatment discontinuation before the excursion event; 1 corresponded to a patient experiencing 3 separate SAEs over a period of 83 days before the excursion (this patient had no other excursions). The remaining 7 events occurred in 3 patients (n=1, 1 event; n=2, 3 events, these 2 patients had low serum drug concentrations during their primary evaluation periods). No patients who experienced serum-free C5 excursions had a treatment-emergent antidrug antibody-positive response. Only one adult patient from the study cohort exhibited a treatment-emergent antidrug antibody response with low antidrug antibody titers and nonneutralizing activity; no free C5 excursions were reported for this patient.

No excursions occurred in the pediatric switch cohort. Excursions occurred in 4 C5i-naïve pediatric patients (1 event each), and all coincided with low serum ravulizumab concentration (<175 μg/mL). One of these 4 events occurred in a patient in the ≥20 kg and <30 kg group and coincided with an extended period of treatment discontinuation. As described previously,^16^ the outlier on day 15 (Fig 6C; serum-free C5 concentration 0.999 μg/mL at predose time point) was noted in a patient from the ≥5 kg and <10 kg group, and this patient received a loading dose of 300 mg per the original dosing regimen prescribed for this weight group (the loading dose was later increased [protocol permitted] to 600 mg based on pharmacokinetic/pharmacodynamic model-based analyses). For both patients who had excursions on day 15, complete inhibition was achieved and maintained in all subsequent pharmacodynamic observations while on the updated maintenance dosing regimen, and corresponding pharmacokinetic C_trough_ values reached therapeutic level. No patients had a treatment-emergent antidrug antibody-positive response.

## Discussion

These results from a 2-year analysis of phase 3 trial data demonstrated that the improved clinical outcomes and quality of life benefits achieved in adult and pediatric patients with aHUS treated with ravulizumab are maintained long-term. Further, the immediate C5 inhibition observed after the first infusion, as reported previously,^11^^,^^12^^,^^16^^,^^17^ was sustained over 2 years with maintenance dosing every 4-8 weeks.^16^^,^^18^

The proportions of patients achieving a complete TMA response increased from week 26 to 2 years (C5i-naïve adults, 54% to 61% and pediatric patients 75% to 90%).^16^^,^^18^ In addition, improvements in hematologic outcomes, kidney function, and quality of life (FACIT-Fatigue score) with ravulizumab treatment were sustained over 2 years.

The majority of C5i-naïve adults and pediatric patients experienced an improvement in eGFR at 2 years compared with baseline. The switch cohort showed a slight decrease in the average eGFR. The decrease was driven by a few patients in this cohort. No pediatric patients in the switch cohort had free C5 excursions during this study period, indicating that eGFR change was not likely because of the pharmacokinetics/pharmacodynamics of ravulizumab. The largest decreases in eGFR were associated with 2 patients whose eGFR subsequently improved. Neither decrease was associated with AEs or changes in other clinical parameters, such as platelet count and LDH. These deviations may reflect changes in eGFR that can be observed among pediatric patients during longitudinal follow-up over the course of their disease and related to minor illnesses and hydration status.

The safety data over 2 years indicate that the majority of AEs and SAEs occurred during the initial evaluation period of 26 weeks.^16^, ^17^, ^18^, ^19^ The higher number of SAEs during the initial evaluation period likely reflects the acute illness that led to manifestation of TMA. Further, the subsequent decrease in SAEs is reassuring.

The safety and tolerability of ravulizumab in this study was similar to the established safety profile of eculizumab in pediatric and adult patients.^21^^,^^22^ Deficiency of the terminal complement pathway may predispose individuals to infection with encapsulated organisms such as meningococcus.^23^^,^^24^ In this study, no meningococcal infections were reported. Meningococcal infections have been observed in other studies of eculizumab and ravulizumab treatment of aHUS or paroxysmal nocturnal hemoglobinuria.^6^^,^^25^^,^^26^ However, all patients with meningococcal infections recovered, and the pharmacovigilance analysis with eculizumab demonstrated that the meningococcal infection rate decreased over time and related mortality remained steady.^26^ This highlights the importance of continued attention to meningococcal vaccinations and antibiotic prophylaxis in this patient population for prevention of meningococcal infections.

The limitations of this study included small sample sizes for some of the cohorts, particularly pediatric switch patients. Small sample sizes could allow the data set to be more sensitive to outliers or skewed distributions. The limited genetic data available for patients, and lack of analysis of outcomes considering genetics, restrict the interpretation of data reported in this study. However, availability of genetic data was driven by patient consent.

Long-term treatment with ravulizumab was well tolerated and associated with continual improvement in TMA response and kidney function in adults and pediatric patients with aHUS. These 2-year data support the favorable benefit/risk profile of ravulizumab and its long-term use in all patients with aHUS.
