# Supplementary material for: Ravulizumab in Atypical Hemolytic Uremic Syndrome: An Analysis of 2-Year Efficacy and Safety Outcomes in 2 Phase 3 Trials
Source: Kidney Med. 2024 Jun 14;6(8):100855. doi: 10.1016/j.xkme.2024.100855 (PMC11298908; doi:10.1016/j.xkme.2024.100855)
Supplement: Supplementary File (PDF) — Figure S1-S2; Table S1-S5. [file mmc1.pdf]

Figure S1. eGFR levels over 2 years in pediatric switch patients.

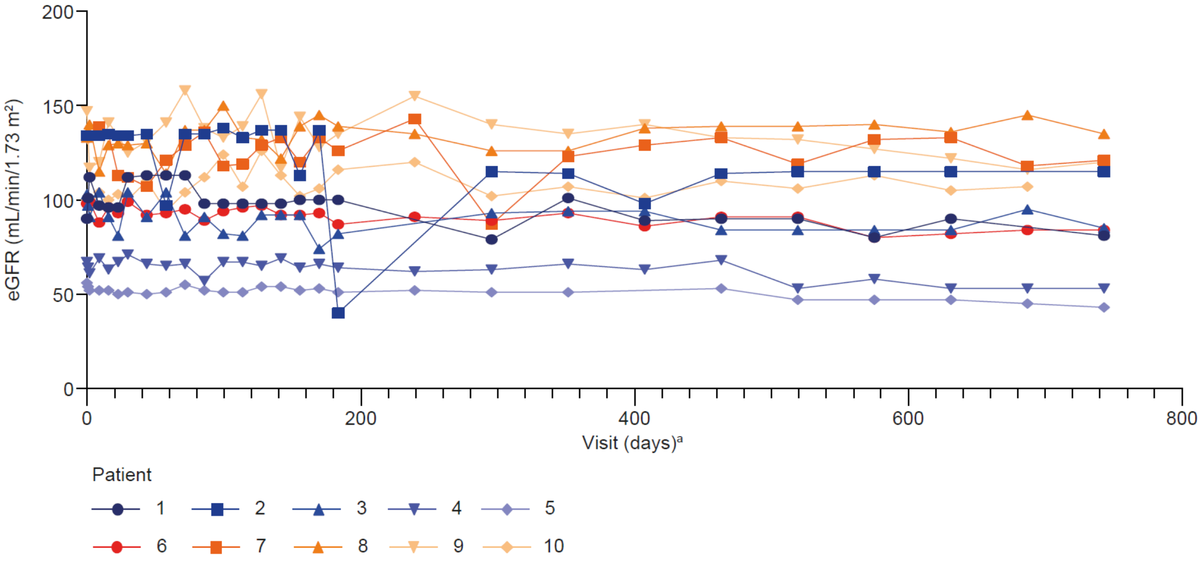

Abbreviation: eGFR, estimated glomerular filtration rate.

[a]The baseline value is defined as the average of the values from the assessments performed prior to the first study drug infusion (this can include results from screening and Day 1 visits). For some data points the patient could not make the scheduled visit and the eGFR reading was taken at the next scheduled visit. Screening visit represented as –1, baseline visit represented at Day 0.

Figure S2. FACIT-Fatigue up to 2 years for adults naive to C5i treatment (A), pediatric patients naive to C5i treatment (B) and pediatric switch patients[a] (C).

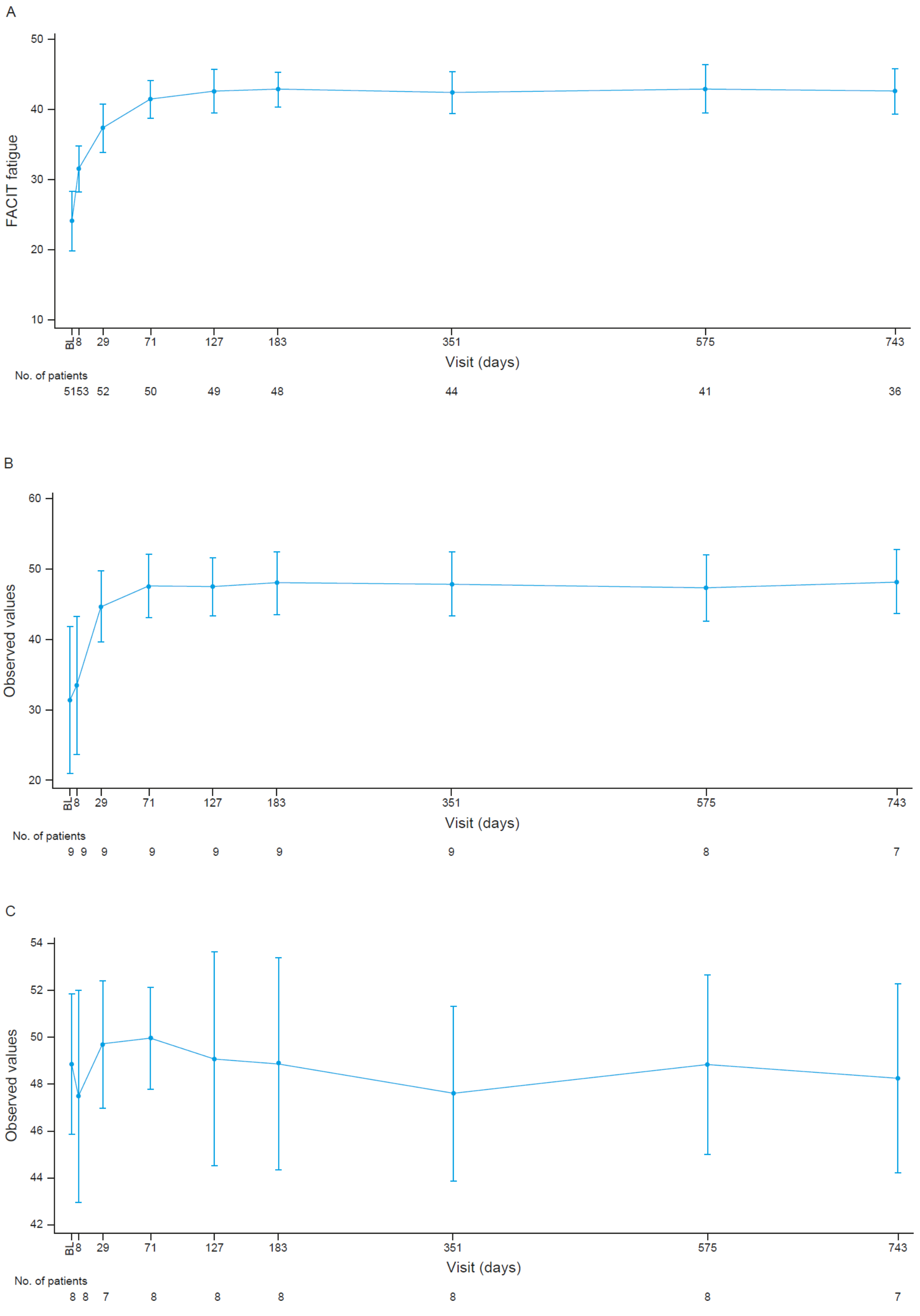

[a]Pediatric FACIT-Fatigue used for children. Data shown as mean (error bars, 95% confidence interval). Abbreviations: BL, baseline; FACIT, Functional Assessment of Chronic Illness Therapy

Table S1. Patient genetic data among patients that entered the extension

C5i-naive adults			
**Patient[a]**	**Clinician genetics findings summary[a,b]**	**Trial genetics/CFH antibody testing[c]**	**Classification[d]**
**1**	N/A	Not tested	N/A
**2**	No pathogenic variant	No pathogenic variant	No pathogenic variant
**3**	*CD46*	*CD46* c.604C>T (p.Leu202Phe)	Likely pathogenic variant in *CD46*
**4**	N/A	*CFB* 6:31950377_A/G p.Lys533Arg	Pathogenic variant in *CFB*
**5**	N/A	Not tested	N/A
**6 KTx**	N/A	No pathogenic variant	No pathogenic variant
**7**	No pathogenic variant	No pathogenic variant	No pathogenic variant
**8**	*CFHR5* c.485_486insAA; p.Glu163Lysfs*10	No pathogenic variant	No pathogenic variant
**9**	N/A	No pathogenic variant	No pathogenic variant
**10**	N/A	No pathogenic variant	No pathogenic variant
**11**	No pathogenic variant, CFH autoantibodies not tested	CFH autoantibodies	No pathogenic variant CFH autoantibodies present
**12**	No pathogenic variant	No pathogenic variant	No pathogenic variant
**13**	No pathogenic variant	No pathogenic variant	No pathogenic variant
**14**	N/A	Not tested	N/A
**15**	N/A	Not tested	N/A
**16**	N/A	No pathogenic variant	No pathogenic variant

**KTx**			
**17**	No pathogenic variant	Not tested	No pathogenic variant
**18**	*CD46* c.649A>G p.(Ser217Gly) homozygous	CFH autoantibodies; genetics not tested	Likely pathogenic variant in *CD46*<br>CFH autoantibodies present
**19**	No pathogenic variant	Not tested	No pathogenic variant
**20**	No pathogenic variant	No pathogenic variant	No pathogenic variant
**21**	N/A	No pathogenic variant	No pathogenic variant
**22**	No pathogenic variant	No pathogenic variant	No pathogenic variant
**23**	No pathogenic variant	No pathogenic variant	No pathogenic variant
**24**	No pathogenic variant	No pathogenic variant	No pathogenic variant
**25**	N/A	*CFHR3* 1:196790152_C/T; rs138675433	No pathogenic variant
**26**	No pathogenic variant	Not tested	No pathogenic variant
**27**	*CFH* c.3486del; p.Lys1162AsnfsTer7	*CFH* c.3226C>G; p.Gln1076Glu<br>*CFH* c.3486del; p.Lys1162AsnfsTer7	Pathogenic variant in *CFH*
**28**<br>**KTx**	N/A	Not tested	N/A
**29**	N/A	Not tested	N/A
**30**	N/A	No pathogenic variant	No pathogenic variant
**31**	N/A	No pathogenic variant	No pathogenic variant
**32**	N/A	No pathogenic variant	No pathogenic variant

**33**	*CD46* c.286+2T>G	*CD46* c.286+2T>G	Pathogenic variant in *CD46*
**34**	N/A	*CFH* c.3691del; p.Arg1231AspfsTer40	Pathogenic variant in *CFH*
**35**	N/A	*C3* c.481C>T; p.Arg161Trp	Pathogenic variant in *C3*
**36**	*CFH* p.Trp314Arg (c.940T>C) heterozygous	Not tested	Pathogenic variant in *CFH*
**37**	No pathogenic variant	No pathogenic variant	No pathogenic variant
**38**	N/A	*CD46* c.175C>T; p.Arg59Ter	Pathogenic variant in *CD46*
**39**	No pathogenic variant	No pathogenic variant	No pathogenic variant
**40 KTx**	N/A	No pathogenic variant	No pathogenic variant
**41**	No pathogenic variant	Not tested	No pathogenic variant
**42**	N/A	No pathogenic variant	No pathogenic variant
**43 KTx**	N/A	No pathogenic variant	No pathogenic variant
**44**	N/A	No pathogenic variant	No pathogenic variant
**45 KTx**	N/A	Not tested	N/A
**46**	N/A	Not tested	N/A
**47**	N/A	Not tested	N/A
**48**	*CFH/CFHR1* fusion gene; *SCR4* and *SCR5* of *CFHR1* replacing *SCR19* and *SCR20* in	No pathogenic variant detected	Pathogenic variant in *CFH*

	CFH		
**49**	CFH deficiency, but no genetics done	*CFH* (NM_000186.4) c.341G>A (p.Cys114Tyr)	Pathogenic variant in *CFH*
**C5i-naive pediatric patients**			
**Patient**	**Clinician genetics findings summary**[b]	**Trial genetics/CFH antibody testing**[c]	**Classification**[d]
**1**	No pathogenic variant; Full report not provided	Not tested	No pathogenic variant
**2**	No response from clinician	Not tested	N/A
**3**	No response from clinician	No pathogenic variant detected	No pathogenic variant
**4**	Homozygous *CFHR1* del and *CFHR3/4* fusion	No pathogenic variant detected<br>CFH autoantibodies	No pathogenic variant<br>CFH autoantibodies present
**5**	*CFH*: A heterozygous c.3593A>T (p.Glu1198Val) in *CFH (*NM_000186.3) It is a likely pathogenic variation (*PM1, PM5, PP5, PM2*)	Heterozygous *CFH* NM_000186.3) c.3593A>T (p.Glu1198Val) found	Likely pathogenic in *CFH*:
**6**	CFH antibodies	No pathogenic variant detected<br>CFH autoantibodies	No pathogenic variant<br>CFH autoantibodies present
**7**	*CFH:* Two pathogenic variants of CFH gene (exon 23):	Not tested	Pathogenic variant in *CFH*:

	c.3572C>G, (p.Ser1191Leu) and c.3590T>C, (p.Val1197Ala, rs460184		
**8**	*MCP*: Pathogenic variant: *MCP* (CD46) heterozygous variant (exon 3) c.493C>T; p.Pro165Ser; rs759136081,	Not tested	Pathogenic variant in *MCP*
**9**	*CFHR* deletion, CFH antibodies; report not provided	Nonsense variant c.958C>T/p.Gln320Ter found on *THBD* gene with NGS<br>CFH autoantibodies	Likely pathogenic in *CFHR*<br>CFH autoantibodies present
**10**	Homozygous *CFHR1* deletion, VUS *C3:* The variant c.4855A>C, p.Ser1619Arg (chr19:6678030:T>G) is located in exon 41 of the gene C3. It is predicted pathogenic by 3 out of 6 available pathogenicity algorithms, GERP++RS (conserved, score: 3.96), Polyphen2 HDIV (possibly damaging)	Missense *VUS* c.4855A>C/p.Ser1619Arg detected by NGS<br>CFH autoantibodies	Variant of uncertain significance in *C3*<br>CFH autoantibodies present
**12**	No response from clinician	Not tested	N/A
**13**	No response from clinician	Not tested	N/A
**14**	No response from clinician	No pathogenic variant detected	No pathogenic

		CFH autoantibodies	variant<br>CFH autoantibodies present
**15**	No response from clinician	No pathogenic variant detected	No pathogenic variant
**16**	No response from clinician	No pathogenic variant detected<br>CFH autoantibodies	No pathogenic variant<br>CFH autoantibodies present
**17**	Compound heterozygous nonsense variants found on gene CD46 (*MCP*): c.381T>A (p.Cys127Ter) and c.685C>T (p.Arg229Ter)	Two pathogenic nonsense variants were detected on gene *CD46* with NGS: c.685C>T (p.Arg229Ter), and c.381T>A (p.Cys127Ter)	Likely pathogenic in *CD46*
**Pediatric switch patients**			
**Patient**	**Clinician genetics findings summary[b]**	**Trial genetics/CFH antibody testing[c]**	**Classification[d]**
**1**	Missense mutation K350N on exon 8 of *CFB* gene	Not tested<br>Negative CFH test	Pathogenic variant in *CFB*
**2**	No pathogenic variant	Not tested<br>Negative CFH test	No pathogenic variant
**3**	Missense mutation Y189D on SCR3 domain of *MCP* gene	Not tested<br>Negative CFH test	Pathogenic variant in *MCP*
**4**	No pathogenic variant	Not tested<br>Negative CFH test	No pathogenic variant
**5**	Not tested	Nonsense variant c.175C>T	Pathogenic in *MCP*,

		(p.Arg59Ter) in exon 2 (SCR1 domain) of member cofactor protein (*MCP*, *CD46*) gene<br>Negative CFH test	*CD46*
**6**	No pathogenic variant	No pathogenic variant detected<br>Negative CFH test	No pathogenic variant
**7**	Missense variant c.3644G>A,p.Arg1215Gin on *CFH* gene	Missense variant c.3644G>A (p.Arg1215Gln) on exon 22 (SCR20 domain) of *CFH* gene<br>Negative CFH test	Pathogenic in *CFH*
**8**	Heterozygous missense variant c.3124C>G (p.Arg1042Gly) on *C3* gene	Not tested<br>Negative CFH test	Pathogenic in *C3*
**9**	No response form clinician	Not tested<br>Negative CFH test	N/A
**10**	No response form clinician	No pathogenic variant detected. CFH autoantibodies	No pathogenic variant<br>CFH autoantibodies present

Abbreviations: CFH, complement factor H, N/A, not available.

[a]Kidney transplant patients are indicated by 'KTx'. [b]Data collected outside of this clinical trial (NCT02949128/NCT03131219) and provided by investigators as response to petition by the trial sponsor for patients who consented. [c]Genetic testing carried out in trial among patients who consented; CFH classification based on either clinician genetics findings and/or this trial (NCT02949128/NCT03131219). [d]Classification based on either clinician genetics findings and/or the trial findings.

Table S2. Renal and hematological outcomes in adults and pediatric patients naive to C5i treatment through 2-year analysis or end of study (full analysis set)

	**Adults naïve to C5i treatment (*n* = 56)**	**Pediatric patients naïve to C5i treatment (*n* = 20)**
**Platelet count normalization, *n* (%)**	48 (86)	19 (95)
**LDH normalization, *n* (%)**	49 (88)	19 (95)
**Hematologic normalization[a], *n* (%)**	48 (86)	19 (95)
**Hemoglobin response[b], *n* (%)**	45 (80)	18 (90)
**≥25% improvement in serum creatinine, *n* (%)**	35 (63)	18 (90)
**Change in eGFR[c] (mL/min/1.73m^2), median (range)**	35 (–6, 95)	82.5 (5, 149)
**Dialysis discontinuation from baseline[a], *n/m* (%)[d]**	12/18 (67)	6/6 (100)
**Dialysis initiation from baseline[b], *n/m* (%)[d]**	4/20 (20)	0/9 (0)

Abbreviations: C5i, complement C5 inhibitor; eGFR, estimated glomerular filtration rate; LDH, lactate dehydrogenase.

[a]Includes normalization of platelet count and normalization of LDH.

[b]Increase in hemoglobin of ≥ 20 g/L from baseline.

[c]Change in eGFR from baseline was calculated using the modification of diet in renal disease formula for adults and the Schwartz formula for children.

[d]*m* refers to the number of patients who were available at the Year 2 (Day 743) visit.

Table S3. Most common[a] adverse events in adults and pediatric patients naive to C5i treatment up to 2 years (safety analysis set)

**Adverse events, *n* (%)**	**Adult patients naive to C5i treatment (*n* = 58)**
**Headache**	23 (40)
**Diarrhea**	20 (35)
**Nausea**	17 (29)
**Vomiting**	17 (29)
**Arthralgia**	16 (28)
**Hypertension**	14 (24)
**Dyspnea**	12 (21)
**Pyrexia**	12 (21)
**Urinary tract infection**	11 (19)
**Anemia**	10 (17)
**Cough**	10 (17)
**Edema peripheral**	10 (17)
**Constipation**	9 (16)
**Fatigue**	9 (16)
**Nasopharyngitis**	9 (16)
	**Pediatric patients naive to C5i treatment (*n* = 24)**
**Pyrexia**	13 (54)
**Diarrhea**	8 (33)
**Vomiting**	8 (33)
**Headache**	7 (29)
**Nasopharyngitis**	7 (29)
**Abdominal pain**	6 (25)
**Hypertension**	6 (25)

**Constipation**	5 (21)
**Contusion**	5 (21)
**Cough**	5 (21)
**Myalgia**	4 (17)
**Nausea**	4 (17)
**Rash**	4 (17)
**Rhinorrhea**	4 (17)
**Upper respiratory tract infection**	4 (17)

Abbreviation: C5i, complement C5 inhibitor.

[a]Defined as any adverse event affecting ≥15% of patients in either group through 2-year follow-up.

Table S4. Most common[a] adverse events in pediatric switch patients up to 2 years (safety analysis set)

	**Pediatric switch patients (n = 10)**
**Upper respiratory tract infection**	4 (40)
**Oropharyngeal pain**	3 (30)
**Pharyngitis**	3 (30)
**Cough**	2 (20)
**Dehydration**	2 (20)
**Gastroenteritis**	2 (20)
**Nasopharyngitis**	2 (20)
**Otitis media**	2 (20)
**Viral upper respiratory tract infection**	2 (20)

[a]Defined as any adverse event affecting ≥15% of patients.

Table S5. SAEs related to infections and infestations

**System organ class**<br><br>Preferred term	***n* (%)**[a]	**Event, *n***
**C5i-naïve adults**		
**Overall infections and infestations**	**16 (27.6)**	**32**
Pneumonia	5 (8.6)	6
Septic shock	2 (3.4)	2
Urinary tract infection	2 (3.4)	4
Device related infection	1 (1.7)	1
Enterococcal infection	1 (1.7)	1
Enterocolitis infectious	1 (1.7)	1
Escherichia pyelonephritis	1 (1.7)	1
Fungaemia	1 (1.7)	1
Gastroenteritis	1 (1.7)	1
Gastroenteritis viral	1 (1.7)	1
Gastrointestinal infection	1 (1.7)	1
Influenza	1 (1.7)	1
Large intestine infection	1 (1.7)	1
Peritonitis	1 (1.7)	2
Peritonitis bacterial	1 (1.7)	1

Pharyngitis	1 (1.7)	1
Pyelonephritis	1 (1.7)	1
Respiratory tract infection	1 (1.7)	1
Sepsis	1 (1.7)	1
Sinusitis	1 (1.7)	1
Staphylococcal sepsis	1 (1.7)	1
Stenotrophomonas infection	1 (1.7)	1
**C5i-naive pediatric patients**		
**Overall infections and infestations**	**9 (37.5)**	**15**
Gastroenteritis rotavirus	2 (8.3)	2
Bronchitis	1 (4.2)	1
Cytomegalovirus enteritis	1 (4.2)	1
Escherichia bacteremia	1 (4.2)	1
Escherichia pyelonephritis	1 (4.2)	1
Gastroenteritis	1 (4.2)	1
Gastroenteritis rotavirus	1 (4.2)	1
Human bocavirus infection	1 (4.2)	1
Pharyngitis	1 (4.2)	1
Pneumonia	1 (4.2)	1
Upper respiratory tract infection	1 (4.2)	1

Viral infection	1 (4.2)	1
Viral pharyngitis	1 (4.2)	2
**Pediatric switch patients**		
**Overall infections and infestations**	**1 (10.0)**	**5**
Bronchitis	1 (10.0)	1
Pneumonia	1 (10.0)	1
Upper respiratory tract infection	1 (10.0)	3

[a]In summarizing *n* (%), if a patient had multiple events for a particular system organ class or preferred term, they were counted only once. Patients may be counted in more than one system organ class or preferred term category.

[b]Patient proportions are based on the total number of patients.
